# The Dynamic of the Apical Ectoplasmic Specialization between Spermatids and Sertoli Cells: The Case of the Small GTPase Rap1

**DOI:** 10.1155/2014/635979

**Published:** 2014-02-27

**Authors:** Giovanna Berruti, Chiara Paiardi

**Affiliations:** Department of Biosciences, University of Milan, 20133 Milano, Italy

## Abstract

Despite advances in assisted reproductive technologies, infertility remains a consistent health problem worldwide. Spermiation is the process through which mature spermatids detach from the supporting Sertoli cells and are released into the tubule lumen. Spermiation failure leads to lack of mature spermatozoa and, if not occasional, could result into azoospermia, major cause of male infertility in human population. Spermatids are led through their differentiation into spermatozoa by the apical ectoplasmic specialization (aES), a testis-specific, actin-based anchoring junction restricted to the Sertoli-spermatid interface. The aES helps spermatid movement across the seminiferous epithelium, promotes spermatid positioning, and prevents the release of immature spermatozoa. To accomplish its functions, aES needs to undergo tightly and timely regulated restructuring. Even if components of aES are partly known, the mechanism/s through which aES is regulated remains still elusive. In this review, we propose a model by which the small GTPase Rap1 could regulate aES assembly/remodelling. The characterization of key players in the dynamic of aES, such as Rap1, could open new possibility to develop prognostic, diagnostic, and therapeutic approaches for male patients under treatment for infertility as well as it could lead to the identification of new target for male contraception.

## 1. Introduction

Spermatogenesis is a very complex and regulated process during which the diploid spermatogonia divide and differentiate into haploid spermatozoa [1–4].

The correct development of fertile spermatozoa relies on the peculiar organization of the seminiferous epithelium. The germinal component (spermatogonia, primary and secondary spermatocytes, round spermatids, and elongating/elongated spermatids) is strictly interconnected with the somatic component, the Sertoli cells, which sustains spermatogenesis giving structural support and nourishment to germ cells [5, 6]. In mouse adult testis, the germ cells at different stages of differentiation display a unique pattern of association with Sertoli cells which can be classified into twelve stages (from I to XII) [1, 2, 4].

**Figure 1:**
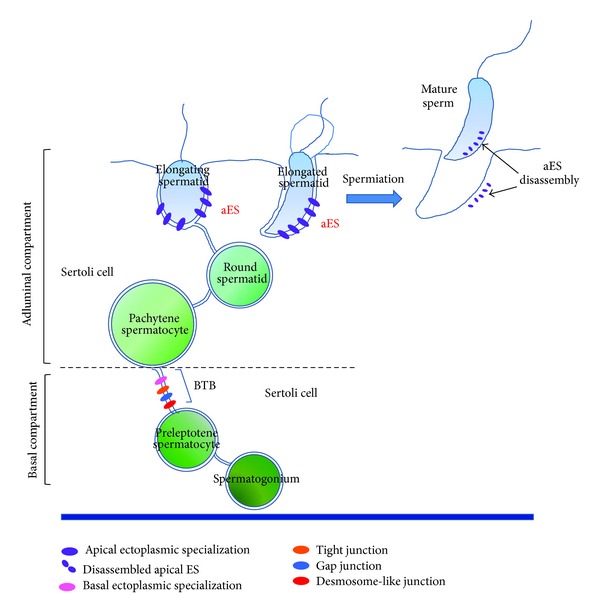
A schematic drawing showing the junctions between Sertoli-Sertoli cells and Sertoli-germ cells as discussed in the review. The drawing illustrates two Sertoli cells embracing germ cells at different stages of differentiation. The Sertoli-Sertoli cell contacts, namely tight junction, gap junction, basal ectoplasmic specialization and desmosome-like junction, give rise altogether to the blood-testis barrier (BTB). The BTB divides the seminiferous epithelium into basal and adluminal compartments. In this last, the apical ectoplasmic specialization (aEs) is established; aEs is the unique type of anchoring junction between Sertoli cell and elongating/elongated spermatids. It undergoes cycles of assembling/disassembling. At spermiation, it disassembles definitely to allow sperm release.

During these stages, spermatids undergo massive morphological modifications such as acquisition of cell polarity, condensation of chromatin, formation of the acrosome and tail, and production and elimination of the residual body. Meanwhile the differentiation process takes place, spermatids migrate across the entire length of the seminiferous epithelium until they reach the luminal edge where mature sperms are finally released [1, 7]. So, it is clear that germ cells have to remain anchored to Sertoli cells till the final steps in order to avoid a premature release as immature spermatids, with consequence on the male fertility potential (Figure 1).

The integrity of seminiferous epithelium and the functional cell interconnections are maintained through several junctional devices that take place between both the Sertoli-Sertoli cells and the Sertoli-germ cells. Besides junction types present also in other epithelia, like tight junctions [8–10] and gap junctions [11, 12], the seminiferous epithelium exhibits testis-unique anchoring junctions, as the ectoplasmic specialization (ES) [13, 14] and the desmosome-like junction [15].

The ES between the Sertoli cells is known as basal ES (bES). At the basal compartment (Figure 1), the bES coexists with other junctional structures like tight junction, gap junction, and desmosome-like junction; all together contribute to create the blood-testis barrier (BTB). The BTB physically divides the seminiferous epithelium in two compartments, that is, a basal compartment where spermatogonia and spermatocytes reside, and an adluminal compartment where spermatids differentiate to develop into spermatozoa (Figure 1) [16, 17]. The establishment of the BTB is fundamental for a successful spermatogenesis; its integrity has to be maintained throughout the entire spermatogenesis [18, 19]. The BTB provides in fact an immunological barrier to the developing male germ cells: it sequesters postmeiotic germ cells from the systemic circulation, thus preventing the production by the host of antibodies against spermatid-specific antigens whose expression is restricted to spermiogenesis only [20]. The BTB likely functions also as a gatekeeper, enabling only the passage through the seminiferous epithelium of selected substances/molecules of support to germ cells. The molecular components and the functions of the BTB have been extensively reviewed (for excellent reviews, see [12, 17, 21]); it will be no longer discussed here.

Conversely, this brief review will focus around the apical ES (aES), restricted to Sertoli-postmeiotic germ cells at the adluminal compartment. Differently from bES, aES does not coexist with other junctions: the aES is the only junctional device that sustains the association between Sertoli and elongating/elongated spermatids (from step 8 of differentiation) until the early phase of spermiation when it disassembles (Figure 1) [13, 22–24].

## 2. Apical Ectoplasmic Specialization

**Figure 2:**
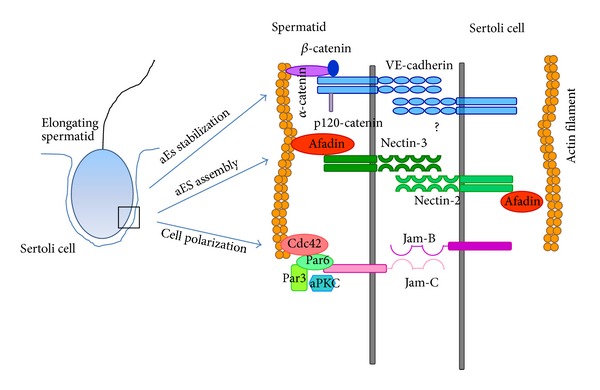
Molecular components at aES level. The picture shows junctional molecules and associated proteins present at aES as discussed here; attention is particularly devoted to junctional complexes on the spermatid membrane. As suggested in the text, an initial Sertoli-spermatid contact may be established by nectins engagement, and then VE-cadherin/catenin complexes contribute to the aES stabilization. In accordance with the literature, the adhesion molecule Jam-C could be involved in spermatid polarization/positioning by recruiting the Par6/Par3/aPKC/Cdc42 polarity complex.

The aES is the best known anchoring junction in the testis. Several studies have led to a significant improvement of our knowledge about its molecular architecture and molecular components. On the basis of such studies, the aES is emerged as a peculiar anchoring junction, being formed by structural components generally found in somatic adherens junctions (the cadherins/catenins and nectins/afadin complexes), tight junctions (such as Jam-C molecules), and focal contacts (the integrin/laminin complex) (Figure 2) [14, 25, 26]. This high heterogeneity is thought to permit that the aES accomplishes its multifunctional role in supporting spermiogenesis. In details, the aES makes the migration possible, concomitantly with the differentiation of spermatids across the seminiferous epithelium. Moreover, the aES could contribute to positioning elongating spermatids with their heads pointed towards the basal compartment. It is to notice that aES is first assembled within the seminiferous epithelium exactly when spermatids begin to loss their spherical shape to become a polarized cell (stage 8) [13, 14, 25]. Finally, the aES maintains spermatids attached to Sertoli cells until these are differentiated and ready to be released into the lumen [13, 23, 24]. It follows that aES has to undergo rapid cycles of assembly and disassembly, concomitantly with the progression of spermiogenesis, and that at spermiation the breakage at cell-cell contacts is definitive. The dynamic of these cycles must be finely and timely regulated. Despite many efforts, the mechanisms governing the aES remodeling during spermiogenesis/spermiation remain however obscure.

Herein, we address attention on how aES dynamic could be regulated. In particular, taking into consideration new experimental evidence provided independently from more laboratories, we discuss a model of regulation of aES assembly/disassembly upon the grounds of findings we obtained from an animal model that we generated appositely to inactivate the small Ras-like GTPase Rap1 [27]. Importantly, it is to underline that this model provides the first genetic link between Rap1 defects and male infertility; the Rap1[S17N] mutation is resulted to be instrumental in revealing the cAMP-Epac-Rap1/extracellular signal-regulated pathway that governs the spermatid-Sertoli cell adhesion at aES. Moreover, it is worth of mention to recall attention on the fact that the central cell of our model is the differentiating spermatid. So far, most works about the aES regulation highlight putative mechanisms operating in Sertoli cells, relegating spermatids to a role of merely spectators. However, the “actors” that are involved in cell-to-cell contact are at least two. Consequently, our model offers a new point of view to investigate about aES dynamic, that is, to consider that also germ cells could be actively engaged.

## 3. Rap1 Regulates aES Dynamic: Experimental Evidence

Rap1 is a member of the family of Ras-like small G proteins [28]; accordingly, it switches between an active conformation bound to the GTP and an inactive one bound to the GDP. The cycle between the two alternative states is coordinated by the guanine nucleotide exchange factors (GEFs), the activators that allow the binding with GTP, and the GTPase activating proteins (GAPs) that enhance the hydrolysis of bound GTP thus leading to Rap1 deactivation [29, 30]. Rap1 functions as a positional signal and organizer of cell architecture; it follows that this GTPase is placed upstream signalling pathways that regulate diverse cellular processes, including morphogenesis [31, 32], cell differentiation [33], cytoskeletal organization [34], cytokinesis [35], exocytosis/endocytosis [36], synaptic plasticity [37], and cell-cell adhesion [30]. As to this last aspect of Rap1 biology, a growing experimental evidence in the last years has allowed to highlight some aspects of Rap1 action in controlling integrin-based as well as cadherin-based junctional systems; in particular, Rap1 has been shown to regulate the levels of E-cadherin and VE-cadherin at the plasma membrane of epithelial and endothelial cells, respectively [38–42].

Rap1 was first detected in the testis in 2000 [43]. It is expressed by germ cells throughout spermatogenesis; in spermatids, in particular, it was immunoprecipitated as a component of the signaling complex formed by the serine-threonine kinase B-Raf and the molecular adaptor 14-3-3 theta protein [43]. To verify the role of Rap1 in vivo in the process of sperm differentiation, we developed a mouse model. Transgenic mice that express a dominant negative mutant of Rap1 (iRap1) were generated; to have the expression of the mutant Rap1 variant selectively only in the postmeiotic germ cells, iRap1 was put under the control of the haploid-specific *Protamine-1* promoter [27]. The phenotype of the mutant mice resulted in a derailment of spermiogenesis due to an anomalous release of immature round spermatids within the tubule lumen and low sperm counts. These findings addressed the research to point up towards the search of Rap1-regulated adhesion molecules leading to the discovery of VE-cadherin and of its epithelial cycle stage specific expression [27].

The high dynamicity of aES renders it as one of the most flexible cell-cell junctions in mammalian tissue, maybe comparable to the better characterized adherens junction at the endothelial cell barrier. This last is known to be a highly dynamic structure; it maintains the integrity of the endothelium, but it is also involved in the control of permeability, leukocyte diapedesis and, more generally speaking, vascular homeostasis [44]. The stabilization of endothelial adherens junctions relies on the stabilization at the plasma membrane of the vascular endothelial cadherin (VE-cadherin) through activation of Rap1 by the cAMP sensor/Rap1-GEF Epac [45]. It is, in fact, widely known that the increase of intracellular cAMP leads to the formation of circumferential actin bundles that support cadherins at adherens junctions; this occurs through Epac-activated Rap1. Similarly, Rap1 is involved in the formation of E-cadherin-based cell-cell adhesions in epithelial cells [38, 40, 41]. However, Aivatiadou et al. [27] found that not only VE-cadherin is expressed in the testis, but it localizes at aES level, exhibiting a pattern of expression that follows aES formation and function; it appears in the adluminal compartment when aESs are being formed and disappears at spermiation, with the exception of the intense staining of the soma of Sertoli cells at the basal compartment. Interestingly, in iRap mutant mice that express a dominant negative Rap1, VE-cadherin is more loosely linked to cytoskeleton and partially tyrosine phosphorylated, two conditions known to be related to impairment of cell-cell adhesions [27, 41]. At last, we remember that the adaptor proteins *α*-, *β*-catenins, essential for linking the cadherins to actin filaments [46], and p120-catenin are expressed in both Sertoli and germ cells [47]. Similarly, germ cells express also afadin [48]. Afadin is a scaffold protein containing an actin-binding and Ras/Rap-binding domain [29, 49] that has been reported to regulate the cyclical activation/inactivation of Rap1 and RhoA [50]. In endothelial and epithelial cells, afadin is involved in Rap1-dependent assembly of cadherins-based adherens junctions (AJs) [40, 51, 52]. The Rap1-GTP/afadin complex mediates the recruitment of p120-catenin to the plasma membrane [51, 52], thus stabilizing VE-cadherin and protecting it from endocytosis [53].

The iRap1 animal model by Aivatiadou et al. [27] clearly demonstrated that Rap1 is involved in the regulation of aES junction dynamic. However, how Rap1 exerts its control and what are its protein targets have not been yet dissected. Very likely, testis VE-cadherin is the adhesion receptor target of Rap1 in spermatids; this, however, does not mean that VE-cadherin is the only one. Here, we suggest, in addition to VE-cadherin, another putative candidate, nectin. Nectins belong to the superfamily of Ca^2+^-independent immunoglobulins that comprises at least four members [49]; they are able to form both homophilic and heterophilic trans-interactions [49]. Nectins have been shown at aESs; more specifically, nectin-2 on the Sertoli membrane trans-interacts with nectin-3 on the spermatid membrane [54]. It has been reported that the nectin-2/nectin-3 complex at aES is stabilized by afadin that connects the complex to the actin filaments [54]. Since afadin could connect with both nectin-based and cadherin-based junctional systems, Rap1 could represent the central regulatory link between these two aES structural complexes. Considering that aESs undergo cycles of assembly and disassembly and that Rap1 mediates both the de novo formation and the reestablishment of adherens junctions [38, 55], the following hypothesis for future experimental research could be suggested. That is, at the assembling, nectins are first engaged at aES recruiting afadin that, on its own, is involved in activation of Rap1; this leads to an accumulation of VE-cadherin at the plasma membrane with the result of strengthening of the junction. The nectins/VE-cadherin adhesion molecules have already been described to be able to connect physically and functionally in endothelial cell systems [56, 57].

## 4. Rap1 as an Organizer of Spermatid Polarization at aES

Rap1 action is accomplished through the cooperation of different signaling pathways involving several effectors [32, 58, 59]. In its central role of positional signal and organizer of cell architecture, Rap1 governs the reorganization of actin cytoskeleton [34, 60, 61]. The great plasticity of the aES requires a rapid rearrangement of actin cytoskeleton; it is not surprising if Rap1 could emerge as a regulator of this cytoskeleton at aES. CDC42 is another Ras-like small GTPase that belongs to the family of Rho-GTPases [62–64], and CDC42 is a Rap1 effector. In epithelial as well as endothelial cells, Epac-activated Rap1 induces CDC42 activation; this leads to a reorganization of actin cytoskeleton resulting in the formation of circumferential actin bundles and consequently in the stability of E-cadherin/VE-cadherin-based cell-cell adhesions [34, 45, 60]. Since CDC42 is expressed in differentiating spermatids [65, 66], it is possible to speculate that Rap1-mediated CDC42 activation is one of the mechanisms that stabilize cell-cell contacts at aESs. Not only, but CDC42 may be the Rap1 effector through which Rap1, acting as a critical regulator of cell polarization [67], controls spermatid polarization, an event for which the contribution of aES has been evoked more times, in the seminiferous epithelium. Cdc42, in fact, is known to work in epithelial cells in concert with the Par-based polarity protein complex to establish the apicobasal cell polarity [68, 69]. Interestingly, in spermatids the Par6/Cdc42/aPKC complex has been shown to be recruited to the plasma membrane by Jam-C, a junctional adhesion molecule found at aES level [65, 70]; Gliki et al. [65] attributed to Jam-C the role of assembling the cell polarity complex and, consequently, of promoting spermatid polarization. However, the pathway though which Jam-C could fulfill such functions was not dissected. In endothelial cells, Jam-C regulates vascular endothelial permeability by modulating VE-cadherin-mediated cell-cell contacts [71]. In particular, the loss of Jam-C expression by Jam-C knockdown results in stabilization of VE-cadherin-mediated adhesion in a Rap1-dependent manner. It follows that the question of Jam-C and Par6/Cdc42/aPKC complex in differentiating spermatids deserves a deeper investigation. Again, Rap1 may emerge as the central regulator that supervises both aES assembly/disassembly and spermatid polarity in a new system, the spermatid-Sertoli cell adhesion system. In this regard, it is to notice that just the VE-cadherin-based AJs have been reported to be essential to establish cell polarity; here, it is in fact recruited the Rap1-activated cell polarity complex in endothelia [72]. So, it is not to exclude that Rap1 could mediate spermatid polarization not only through the Jam-C/polarity complex but also through the VE-cadherin/polarity complex system.

## 5. Rap1, Rho, and the Disassembling of aES

Rho belongs, as CDC42 and Rac1, to the family of Rho GTPases and is involved in the dynamics of F-actin structures thus influencing cell shape and assembly of AJs. The best characterized Rho isoforms are RhoA, RhoB, and RhoC [73, 74]; RhoA and RhoB have been reported in testis and spermatozoa [66]. While CDC42 and Rac1 are known to act as Rap1 effectors, that is, downstream the activated Rap1, Rho GTPase is best known to counteract the action of Rap1 [27, 34, 75]. For example, activation of the Rho GTPase pathway determines endothelial cell hyperpermeability and could lead to endothelial barrier disruption [75, 76]. Accordingly, overexpression of a constitutively active mutant of Rap1 results in activation of Rac1 and, intriguingly, inactivation of RhoA [50]. As already noticed, with few exceptions, members of the Ras-like GTPase super-family cycle, between an active and inactive state. The continuous cycling between the two states is tightly controlled by a number of regulatory proteins that specify where and for how long the signal is “on” and which cellular function is modulated. In the case of cell adhesion, for each cellular process triggered by cell-cell contacts, multiple GTPases must be dynamically turned “on” or “off” [34, 50, 75]. This “turning” is facilitated by GEFs and GAPs, respectively. So far, several GEFs and GAPs have been described to modulate the organization, molecular composition, and function of adhesive complexes in different cell types. In endothelial cells, the second messenger cAMP induces barrier protective responses against thrombin or inflammatory mediators [77, 78]. The Rap1 GEFs that are sensors of cAMP elevation are known as Epac (exchange protein activated by cAMP), of which there are two variants, Epac1 and Epac2 [79–81]. Rap1, however, could be activated also by other GEFs, such as C3G, PDZ-GEFs, and CalDAGs [59, 82, 83]. Still referring to the endothelial cell AJs, Birukova et al. [75] have shown that the Rap1 PDZ-GEF cooperates with Epac to maintain junction integrity; more specifically, the PDZ-GEF is involved mainly in Rap1 activation under resting conditions [45], while Epac/s is/are necessary to further tighten cell-cell contacts [45]. Both Epac1 and Epac2 have been found in mouse testis and male germ cells [80, 84]. Similar to the GEFs, there are various Rap-GAPs that are specifically targeted to different molecular complexes at various cellular locations [59]. These GAPs may reverse the dynamic processes controlled by activated Rap1. Among the best characterized Rap1-GAPs, there are Rap1-GAP1,2, Spa-1, and SPAR1,2,3 [59]; so far, however, experimental evidence for Rap1-GAPs in the testis is still lacking.

This is not the case, on the contrary, for RhoGAPs. Aivatiadou et al. [80] reported that male germ cells express RA-RhoGAP. RA-RhoGAP is a RhoGAP that possesses a RA domain through which it binds to Epac-activated Rap1 thus acting as a Rap1 effector and transductor of signal from Rap1 to Rho [85]. Indeed, Aivatiadou et al. [80] showed also that in isolated spermatogenic cells, after stimulation with 8-CPT (a membrane-permeable analogue of cAMP), Epac-activated Rap1 colocalizes with RA-RhoGAP. It follows that male germ cells have the equipment of signaling molecules, including activators and effectors, through which activated Rap1 could suppress the action of Rho.

At this point, if Rap1 and Rho are likely the Ras-like GTPases that govern aES dynamic, a key question concerns the signal/s responsible for the Rap1/Rho activation/deactivation at aES. Before the conclusion of this brief review, we want to provide a further suggestion towards the direction for this putative signaling molecule. Transforming growth factor-*β*1 (TGF-*β*1) functions in diverse cellular processes, such as tissue differentiation and cell migration. Recent experimental evidence on signaling that governs monocyte adhesion and chemotaxis [86] has revealed that TGF-*β*1 triggers cAMP elevation leading to Rap1 activation via Epac; not only this, but also this Rap1 activation, on its own, results in Rho inactivation through the Rap1-dependent RhoGAP. In other words, prolonged TGF-*β*1-treated cells produce cAMP, which activates sequentially Epac, Rap1, and Rap1-dependent RhoGAP, resulting in suppression of Rho and macrophage migration. TGF-*β*1 is produced in the testis by both germ cells and Sertoli cells [87]. TGF-*β*1 may thus result to be crucial for the restructuring of aES.

**Figure 3:**
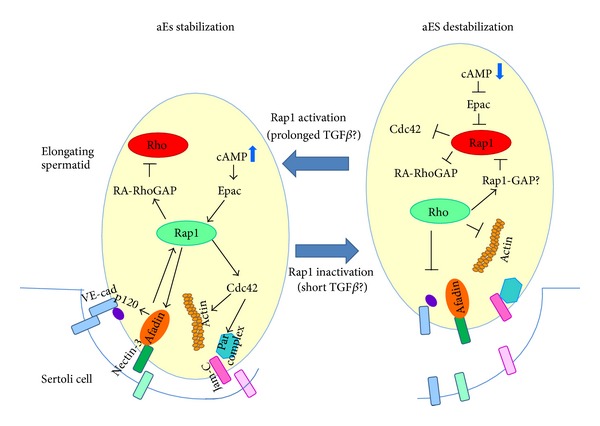
The proposed model of regulation of aES dynamic. The aES structuring/restructuring is governed by a coordinated crosstalk between Ras-like small GTPases, among which Rap1 is the central core. Under conditions that favor Sertoli-spermatid contacts (TGF*β* is here suggested as one of the putative candidates that promotes the cAMP signaling for establishment of cell-cell contact), the triggered cAMP elevation leads to Epac-mediated Rap1 activation; Rap1 action stabilizes aES junctions acting on both nectin/afadin and VE-cadherin/catenin complexes as well as on CDC42 thus promoting actin filament organization. Moreover, activated Rap1 acts on another Rap1 effector, namely, RA-RhoGAP, so that the activity of Rho-GTPase is switched off. Conversely, under conditions that promote aES destabilization (here suggested as a short exposition to TGF*β*), the consequent decrease in cAMP level leads to Rap1 deactivation with the downstream effects on the junction stability and F-actin organization; concomitantly, RA-RhoGAP is not activated and the Rho-driven disassembling of aES could take place. All the proteins discussed here are known to be expressed in male germ cells. See text for details and further discussion.

Conclusively, here we provide a model (Figure 3) for the mechanism/s by which Rap1 could potentially regulate aES dynamic. It is to remark that here we took into consideration deliberately only spermatids, that is, the cells so far rather neglected under this context. Restructuring of aES junction is thought to be dependent on a highly coordinated network of mechanisms of activation/deactivation of Ras-like GTPases that has Rap1 as its central hub and the Rho-family GTPases as downstream Rap1-effectors/antagonists. Rap1 action results in the assembly/stabilization/reestablishment of aES junctions whereas Rho drives their disassembling; interestingly, the functional Rap1/Rho interaction is proposed to be vice-versa reciprocal. Obviously, some aspects of the suggested regulation of aES dynamic need to be verified and are waiting for the experimental validation.

## 6. Conclusion

Male infertility is one of the health problems worldwide. Several defects responsible for male infertility are still unclear, mostly due to our poor understanding of molecular mechanisms that regulate sperm production and release from the testis, the maturation and transit of the sperm through the male and female tracts, and the events essential for fertilization. Because we do not understand entirely these molecular processes, we cannot diagnose correctly the causes of male infertility. Upon the grounds of the phenotype that characterizes the unique animal model developed so far to investigate in vivo about the nature of the signaling involved in the regulation of spermatid-Sertoli cell junctions [27], here we propose two interconnected mechanisms that could unravel the question of the regulation of a highly dynamic and essential junction like the aES is.
